# Hepatitis B Virus Assembly: Cryo‐Electron Microscopy Reveals Structure of the Surface Antigen

**DOI:** 10.1002/mco2.70348

**Published:** 2025-08-27

**Authors:** Guobao Li, Jiaxin Cui, Jifeng Nie

**Affiliations:** ^1^ School of Resources and Environment Baoshan University Baoshan Yunnan China; ^2^ School of Foreign Languages Baoshan University Baoshan Yunnan China; ^3^ Department of Hepatobiliary Surgery Zhejiang Hospital of Integrated Traditional Chinese and Western Medicine Hangzhou China

1

In a recent study published in *Science*, Wang et al. provided novel insights into the assembly of the hepatitis B virus (HBV) by utilizing cryo‐electron microscopy (cryo‐EM) [1]. Through reconstruction of *D*_2_(222)‐ and *D*_4_(422)‐like quasisymmetric subviral particles (SVPs) combined with near atomic‐level determination of HBsAg topology, they elucidated how HBsAg dimers polymerize into higher‐order architectures—enabling SVP formation and coordinated interactions with HBV nucleocapsids to assemble infectious virions.

The World Health Organization (WHO) estimates that approximately 254 million people were living with chronic hepatitis B in 2022. Viral hepatitis caused 1.3 million deaths globally, with HBV accounting for 83% of the fatalities. As a member of the *Hepadnaviridae* family, HBV generates non‐infectious spherical SVPs (17–22 nm in diameter), filamentous SVPs (22 nm in diameter), and infectious Dane particles (∼44 nm in diameter) during its life cycle. Notably, SVPs can outnumber Dane particles by up to 10,000‐fold, contributing to immune tolerance and the persistence of chronic infection. Deciphering the architecture and assembly of HBV particles remains crucial for understanding viral morphogenesis. However, the acquisition of SVPs and their HBsAg‐mediated structural heterogeneity present significant barriers to high‐resolution structural elucidation of distinct SVP subtypes. Elucidating HBsAg conformation is essential for rational vaccine design, neutralizing epitope mapping, and HBV therapeutics development.

HBsAg is translated from a single open reading frame encoding three co‐terminal isoforms: small (S‐), medium (M‐), and large (L‐) HBsAg (Figure 1A). Among these, S‐HBsAg constitutes the predominant component in both SVPs and infectious Dane particles. Wang et al. expressed S‐HBsAg in human embryonic kidney 293 (HEK293) and Chinese hamster ovary (CHO) cells, generating SVPs morphologically analogous to native virions. HEK293‐derived SVPs were purified by affinity chromatography, whereas CHO‐derived SVPs were isolated via sucrose gradient centrifugation. Both populations underwent size‐exclusion chromatography to exclude aggregates and isolate monodisperse particles, ensuring sample homogeneity for cryo‐EM. After data acquisition and analysis, two monodisperse populations of quasi‐spherical SVPs with a diameter of 22 nm were identified. Single‐particle reconstruction revealed two distinct icosahedral symmetry classes: *D*_2_(222) and *D*_4_(422) (referred to as “D2” and “D4,” respectively). By applying symmetry constraints and a custom orientation transformation script, the authors achieved a 3.7 Å resolution structure of S‐HBsAg. It comprises two N‐terminal transmembrane α‐helices (TH1 and TH2) and four C‐terminal membrane‐embedded α‐helices (EH1–EH4) (Figure 1B). The protein integrates into the lipid bilayer with the cytoplasmic loop (CL) between TH1 and TH2 oriented toward particle interior, while the luminal loop (LL) between TH2 and the hydrophobic C‐terminal region protrudes outward (Figure 1B). In these SVPs, HBsAg forms homodimers, with TH1 and EH3 mediating dimer polymerization into higher‐order assemblies: trimeric and tetrameric arrangements of dimers (Figure 1C).

**FIGURE 1:**
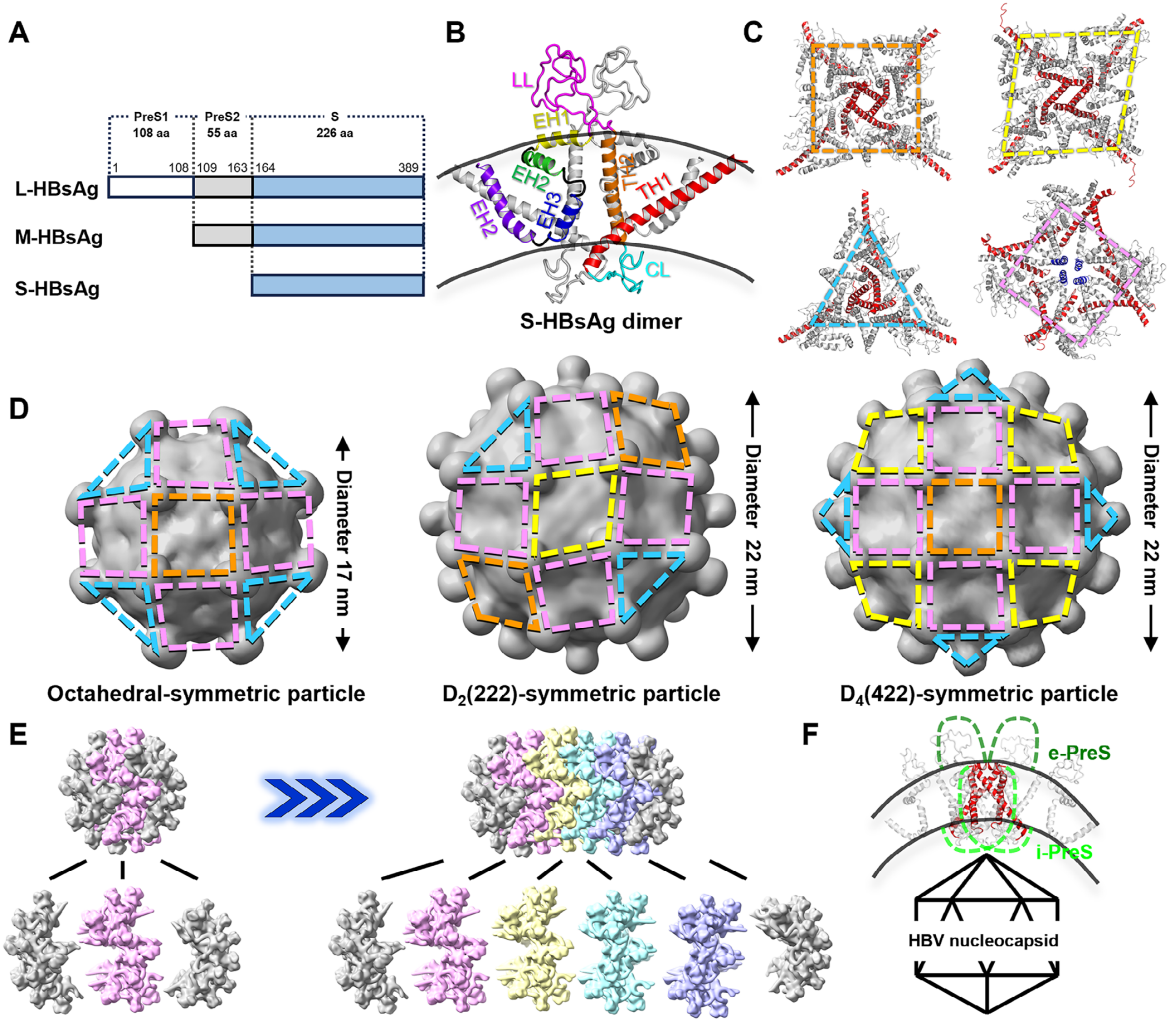
The role of HBsAg in HBV particle assembly. (A) Schematic representation of three HBsAg isoforms: L‐HBsAg (PreS1, residues 1–108, white; PreS2, residues 109–163, gray; S‐domain, residues 164–389, blue), M‐HBsAg (PreS2 + S‐domain), and S‐HBsAg (S‐domain only). Domain boundaries are defined according to UniProtKB entry P03142. (B) Structural topology of the S‐HBsAg dimer. S‐HBsAg contains two transmembrane helices (TH1 and TH2), four membrane‐embedded α‐helices (EH1–EH4), a cytosolic loop (CL), and an ER lumenal loop (LL). Two S‐HBsAg molecules form a homodimer. (C) Higher‐order assembly of S‐HBsAg dimers. S‐HBsAg dimers further assemble into two structural units via TH1 helices (red): a tetramer (orange square and yellow diamond) and a trimer (light blue triangle). An additional tetrameric assembly (pink square) is stabilized by EH3 helices (dark blue). (D) Assembly differences and relationships between octahedral‐, *D*_2_ (222)‐, and *D*_4_ (422)‐symmetric particles. All three particle types are assembled from trimeric and tetrameric HBsAg dimer structural units. The diameters of each particle type are indicated. (E) Proposed assembly mechanism for filament‐shaped SVPs. The D4 particle contains two hemispherical caps (gray) at the poles and a central equatorial belt (pink). The equatorial belt acts as the connecting element, repeating between the cap structures to elongate the particle and generate filamentous particles of varying lengths. (F) In the diamond‐shaped tetramer formed by HBsAg dimers, the N‐terminal preS domains of HBsAg are oriented toward the exterior (e‐preS) or interior (i‐preS) depending on the position of TH1 within the membrane. The interior‐oriented i‐preS domains are proposed to interact with the nucleocapsid. This figure was created using PyMOL and ChimeraX.

Despite distinct architectures among D2, D4, and 17‐nm octahedral‐symmetric particles [2], all share a common assembly rule mediated by the trimeric and tetrameric units (Figure 1D). Filamentous particles were additionally observed, exhibiting widths equivalent to the 22 nm diameter of spherical SVPs but with variable lengths. Structural dissection of the D4 particle revealed a tripartite architecture: two hemispherical caps and an equatorial belt‐like module (Figure 1E). The belt‐like module can be extended by stacking additional ones between the two cap‐like structures, thereby enabling the formation of filaments with varying lengths.

Recent structural studies by He et al. have characterized the assembly pattern of M‐HBsAg within SVPs [3]. Unlike S‐HBsAg, which contains only the S domain, M‐HBsAg contains both the PreS2 and S domains (Figure 1A). However, the PreS2 region lacks resolvable density in the cryo‐EM maps, suggesting its structural flexibility compared to the S domain inserted into the SVP surface. Notably, M‐HBsAg‐driven SVPs have a core diameter of ∼17 nm (22 nm with protrusion). This core size is smaller than that of S‐HBsAg‐driven SVPs (core: 22 nm; 28 nm with protrusion) but comparable to octahedral‐symmetric SVPs (core: ∼17 nm; 22 nm with protrusion). Furthermore, M‐HBsAg‐driven SVPs exhibited structural heterogeneity, as shown by non‐uniform electron density in cryo‐EM maps. The authors suggested that biological (e.g., expression systems) and technical variables (e.g., sample preparation, data processing) might contribute to these discrepancies, though the exact mechanisms require further elucidation.

Structural comparison between M‐HBsAg and S‐HBsAg revealed superimposable conformations in the N‐terminal helices (TH1, TH2, and EH1), whereas divergent conformations were observed in the C‐terminal helices (EH2‐4 regions). These findings indicate the intrinsic flexibility of HBsAg, which may facilitate its adaptation to diverse packing microenvironments and provide mechanistic insights into viral assembly. Moreover, He et al. resolved the CL domain's well‐defined architecture, stabilized by a CHC2‐type zinc finger motif—a feature conserved across HBV‐related viruses—achieving higher local resolution than previous studies by Wang et al. Disruption of this motif via cysteine‐to‐alanine substitutions abolished HBsAg surface expression, highlighting the zinc finger's essential role in viral antigen biogenesis. Notably, while He et al. proposed a five‐helix model for the HBsAg region, Wang et al. reported a four‐helix structure. The C‐terminal helices H5b and H5c in He et al.’s model spatially correspond to the single EH4 helix in Wang et al.’s model, with the H5b/H5c region exhibiting slightly higher curvature. This discrepancy in helix enumeration likely reflects advancements in cryo‐EM resolution rather than intrinsic differences between M‐ and S‐HBsAg isoforms. He et al.’s M‐HBsAg achieved 3.6 Å resolution with improved local map quality, critical for definitive helix assignment, while Wang et al.’s S‐HBsAg (3.7 Å) exhibited lower local resolution in these regions.

Non‐infectious 17–22 nm SVPs cannot account for 44 nm Dane particle assembly, which requires icosahedral nucleocapsid incorporation (∼36 nm in diameter) [1, 4]. Nucleocapsid envelopment requires L‐HBsAg, whose N‐terminal residues 92–113 facilitate nucleocapsid docking to packaging sites, a function absent in S‐/M‐HBsAg isoforms. Wang et al. implicated through higher‐order dimer polymerization analyzes that the pre‐TH1 domain of L‐HBsAg exhibits dual membrane topology, accessing either the virion exterior or interior depending on its oligomeric state. In trimeric HBsAg dimers, the N‐termini of the TH1 domain are exposed, whereas in diamond‐shaped tetramers, the N‐termini of the TH1 helices are embedded into the membrane or exposed on the surface (Figure 1F). This structural plasticity drives functional bifurcation: externally oriented L‐HBsAg facilitates viral attachment/entry via exposed N‐termini (Figure 1F), while membrane‐embedded TH1 domains anchor N‐terminal regions near inner‐surface CLs, enabling nucleocapsid interaction. Seitz et al. proposed a maturation‐dependent topological switch: following the dissociation of preS domain‐nucleocapsid interactions, unbound L‐HBsAg undergoes a conformational change, flipping the preS domains from the interior of the particle to the exterior [5]. However, the structural dynamics of domain flipping, and the temporal coordination of these maturation steps remain unclear.

Cryo‐electron tomography (cryo‐ET), a powerful technique capable of resolving dynamic biological processes in their native state, offers unprecedented opportunities to dissect structural dynamics during SVP assembly. By applying cryo‐ET across multiple post‐infection stages, future studies could systematically map the temporal and spatial reorganizations driving viral particle formation. This approach is poised to decode the distinct roles of L‐, M‐, and S‐HBsAg in the molecular orchestration of assembly processes, thereby addressing long‐standing questions about their structural cooperativity. Furthermore, investigating how the lipid environment within the particles influences their assembly may provide critical insights into the role of lipid composition in SVP and virion formation. Such insights would not only advance the fundamental understanding of HBV morphogenesis but also inform the rational design of HBsAg‐targeted therapeutics, potentially disrupting virion assembly or blocking infectivity.

## Author Contributions

G.L. drafted the manuscript and prepared the figure. J.C. and J.N. discussed and revised the manuscript. All authors have read and approved the article.

2

This work was supported by the Ph.D. Research Start‐up Fund of Baoshan University (BSKY202501).

## Ethics Statement

The authors have nothing to report.

## Conflicts of Interest

The authors declare no conflicts of interest.

## Data Availability

The models used for the structural analysis of HBsAg are available in the Protein Data Bank (PDB) under accession codes 8YMK (HBsAg dimer) and 8YMJ (higher‐order assembly of HBsAg dimers). The cryo‐EM density maps used to compare subviral particles are available in the Electron Microscopy Data Bank (EMDB) under accession codes EMD‐39395 (D2 particle), EMD‐39397 (D4 particle), and EMD‐26117 (octahedral‐symmetric particle).

## References

[1] Q. Wang , T. Wang , L. Cao , et al., “Inherent Symmetry and Flexibility in Hepatitis B Virus Subviral Particles,” Science 385, no. 6714 (2024): 1217–1224. 39264996 10.1126/science.adp1453

[2] H. Liu , X. Hong , J. Xi , S. Menne , J. Hu , and J. C. Wang , “Cryo‐EM Structures of Human Hepatitis B and Woodchuck Hepatitis Virus Small Spherical Subviral Particles,” Science Advances 8, no. 31 (2022): eabo4184. 35930632 10.1126/sciadv.abo4184 PMC9355357

[3] X. He , Y. Kang , W. Tao , J. Xu , X. Liu , and L. Chen , “Structure of Small HBV Surface Antigen Reveals Mechanism of Dimer Formation,” Cell Discovery 11, no. 1 (2025): 6. 39805867 10.1038/s41421-024-00768-8 PMC11730374

[4] B. Venkatakrishnan and A. Zlotnick , “The Structural Biology of Hepatitis B Virus: Form and Function,” Annual Review of Virology 3, no. 1 (2016): 429–451. 10.1146/annurev-virology-110615-042238 PMC5646271 27482896

[5] S. Seitz , J. Habjanic , A. K. Schutz , and R. Bartenschlager , “The Hepatitis B Virus Envelope Proteins: Molecular Gymnastics Throughout the Viral Life Cycle,” Annual Review of Virology 7, no. 1 (2020): 263–288. 10.1146/annurev-virology-092818-015508 32600157

